# From Research to Practice: Which Research Strategy Contributes More to Clinical Excellence? Comparing High-Volume versus High-Quality Biomedical Research

**DOI:** 10.1371/journal.pone.0129259

**Published:** 2015-06-24

**Authors:** Anat Tchetchik, Amir Grinstein, Eran Manes, Daniel Shapira, Ronen Durst

**Affiliations:** 1 Department of Business and Management, Guilford Glazer Faculty of Management, Ben Gurion University of the Negev, Beer Sheva, Israel; 2 The School of Industrial Management, Jerusalem College of Technology, Jerusalem, Israel; 3 Faculty of Economics and Business Administration, VU University Amsterdam, Amsterdam, The Netherlands; 4 D’Amore-McKim School of Business, Northeastern University, Boston, Massachusetts, United States of America; 5 Cardiology Division, Hadassah Hebrew University Medical Center, Jerusalem, Israel; VU University Amsterdam, NETHERLANDS

## Abstract

The question when and to what extent academic research can benefit society is of great interest to policy-makers and the academic community. Physicians in university hospitals represent a highly relevant test-group for studying the link between research and practice because they engage in biomedical academic research while also providing medical care of measurable quality. Physicians’ research contribution to medical practice can be driven by either high-volume or high-quality research productivity, as often pursuing one productivity strategy excludes the other. To empirically examine the differential contribution to medical practice of the two strategies, we collected secondary data on departments across three specializations (Cardiology, Oncology and Orthopedics) in 50 U.S.-based university hospitals served by 4,330 physicians. Data on volume and quality of biomedical research at each department was correlated with publicly available ratings of departments’ quality of care, demonstrating that high-quality research has significantly greater contribution to quality of care than high-volume research.

## Introduction

The question when and to what extent academic research can benefit practice is a topic of great interest and debate among policy makers and academic researchers [1–4]. Whereas there are recent calls and policies to encourage universities to play an even more active role in the commercialization of academic knowledge and overall to be more engaged with industry [1], [5–7], in reality the gap between theory or research and capability of transferring its results into practice is repeatedly evidenced [3], [8–9]. Further, within the broad topic of the link between academic research and practice a host of open questions remain, for example, what type of academic research can be most effectively translated into better practice? One key reason is that although of clear and enormous importance, the impact of academic research on practice cannot often be easily quantified. Unlike private enterprises that can measure the marginal effect of their expenditure in R&D on economic profits, most publicly funded research is made by non-profit organizations and most efforts of academics are aimed at a single key output: research publications. That is, the accepted measures of academic research impact on society are based on the research output itself rather than its impact beyond “the ivory tower.”

Improvements in practice may potentially be driven from a research strategy that either boosts high-volume research productivity or promotes high-quality research productivity [10]. While the debate among advocators of these two strategies is not in itself novel, most of the literature has been focusing on the extent by which these competing strategies correspond with scientific excellence, while overlooking their linkage with practice. [11]. On the one hand, proponents of the high-quality strategy argue that greater quantity inevitably comes at the expanse of quality [10–12]. This has been demonstrated, for example, in physics and in biomedical research [13–14]. Within the biomedical research domain, it has been noted by a former editor of the *New England Journal of Medicine* that researchers should be judged primarily by the quality and number of their real scientific contributions, not by the quantity of papers they publish, [15–16]. On the other hand, it is argued that creativity and breakthroughs in science might be the result of numerous trials-and-errors, and that researchers ought to work on a large set of experiments that may result in a large set of minor findings and publications before they eventually establish high-quality research finding. According to this view, therefore, quantity is a necessary (yet insufficient) condition for quality [10], [17–19]. Some empirical findings indeed lend support to this view [10, 20].

This work aims at offering an empirical contribution to the study of the research-practice nexus. Attempts to study this link were limited to the R&D literature and employed indicators such as ‘engagement with industry’ and patenting as a quantifiable proxy for the contribution of R&D to practice [7], [21–25]. Yet, the important linkage between bio-medical research and medical practice has not yet been studied. We attempt at filling this gap by studying physicians in university hospitals. This group represents a highly relevant test case because it engages in biomedical academic research while also providing medical care of measurable quality. Furthermore, biomedical academic research is one of the most heavily funded research areas by governments worldwide. In the United States, for example, at the National Institutes of Health (NIH) in 2012 a budget request for $31.987 billion was aimed at providing funds for more than 40,000 competitive research grants and more than 325,000 research personnel at over 3,000 research institutions and small businesses across the country [26]. Hospital physicians constitute a central group that benefits from these funds: in that year about 60% of the funds awarded by NIH were aimed at medical schools and independent hospitals [27]. Underpinning this policy is the expectation that biomedical academic research makes a valuable contribution to medical practice. Specifically, physicians who engage in biomedical research are expected to improve their clinical capacities because of their exposure to up-to-date medical knowledge and advanced technologies and applications [28]. As physicians’ resource constraints make it difficult to achieve both volume and quality [29–30], there is likely to be a trade-off between the two research strategies, and it is also not at all clear which of the two strategies is of more relevance for healthcare improvement [31–37]. For this reason, and in an effort to maximize the added value of academic research to practice–in the context of biomedical research–the following question arises: what is the type of research productivity strategy that is expected to yield a higher benefit to medical practice? We believe this research question is both novel and important.

While the contribution of biomedical research to medical practice is potentially substantial, any attempt to quantify it runs into both theoretical and methodological difficulties. Nevertheless, such attempts are important if we accept that the justification for generous support of research should be based on measurable performance. For that purpose, we draw a distinction between *direct* and *indirect* contributions of biomedical research, of which the former, as defined next, is both quantifiable and measurable. Publishing a scholarly article that reports the findings of a clinical trial or epidemiologic study may have two main effects, among others: it may help streamline existing treatment and care methods, improve diagnostic procedures and so forth. This is a direct effect as it has a straightforward and immediate impact on the quality of healthcare. The greater the extent to which the study employed high-quality techniques and state of the art methodologies, the bigger is the expected contribution of the study to the quality of care. The second, indirect, effect by which the study may affect the existing body of knowledge is by triggering and inspiring future related studies. In that respect, the overall scientific quality of the article may be less important compared to the speed with which it becomes accessible to the relevant scientific community.

In this study we aimed to examine only the direct differential influence of the high-quality versus the high-volume research productivity on the quality of medical care. Capitalizing on the fact that physicians in university hospitals engage in biomedical academic research while also providing medical care of measurable quality, we aimed at measuring the synergy effect between both activities, thus focusing entirely on the direct contribution of research productivity to the quality of medical care while ignoring indirect channels of potential influence. To this end we collected secondary data on departments across three specializations (Cardiology, Oncology and Orthopedics) in 50 U.S.-based university hospitals served by 4,330 physicians. After controlling for covariates known to influence quality of medical care (e.g., hospital’s income per physician, size, and patients’ characteristics), we examined the correlation between the volume and quality of publications of physicians in each department and two measures of the department’s quality of medical care as reported in *U*.*S*. *News & World Report*’s “Best Hospitals” quality of care ranking.

### Theoretical reasoning and hypothesis

The idea that investment in R&D is a major determinant of output and long-term growth is a well-established paradigm in the literature on R&D and economic growth, where it enjoys wide theoretical and empirical support [38]. Not only biomedical research but academic research in general confers clear and important long-term benefits on society. The advance of science and academic research continues to play a major role in the progress of humanity by raising the stock of knowledge and improving existing techniques and methods in a multiplicity of areas, not the least of them life-saving treatments and medications. Yet, measurement of this overall effect faces obvious obstacles. However, focusing on the direct effect allows us to identify potential *synergy* between research and (medical) practice. The underlying rationale is straightforward: by keeping up with latest advances in related fields and literatures, by attending conferences and maintaining contacts with peers, and by reading (and writing) papers that utilize state-of-the-art techniques and methods in the field, physicians who engage in biomedical research are supposed to greatly improve their capacities in whatever realm of practical medicine they occupy. Based on the generally accepted arguments that compared to low-quality research, high-quality research involves exposure to more advanced techniques, more sophisticated literatures and more cutting-edge methods, and that there is a trade-off between volume and quality [10], [15], [39] we suggest the following hypothesis:

**Hypothesis:** *High-quality research productivity at the department level is associated with greater direct contribution to quality of medical care than high-volume research productivity*.

It is important to note that our results bear little if any relevance to other potential channels through which research may contribute to the quality of health care, such as the indirect effect mentioned above.

## Material and Methods

### Sample

Our original intention was to collect data on all physicians whose names appeared on the websites of the sampled hospital departments. We subsequently decided, however, that in the case of departments with more than 100 physicians a sub-sample of approximately 40 randomly selected physicians would suffice. Our sample included 50 U.S.-based university hospitals engaged in biomedical research (S1 Table). Using a publicly available list of all U.S.-based university hospitals as the sampling frame (N = 133) [40] and a stratified sampling approach, we identified 50 geographically heterogeneous hospitals. Within each hospital we identified three central specializations: Cardiology, Oncology, and Orthopedics. Overall, therefore, our data comprised 150 department-level observations.

### Biomedical academic research: volume versus quality

The two key independent variables were the volume and quality of biomedical academic research conducted by hospital physicians in each of the three specializations at the department level. These data were not readily available and had to be retrieved through a detailed bibliometric database search for each physician (N = 4,330). The individual-level data were then aggregated to yield the department-level data. A detailed description of the construction of the two variables follows.

To estimate the research volume of sampled physicians, PubMed was used as the basis for calculation of the annual average number of publications during the period between 2002 and 2011. To ensure proper identification of the relevant physician, we typed the physician’s full name (surname, first name, middle initial) in the “author field”. Such a search has the disadvantage of retrieving only publications since 2002, whereas a less limiting search (surname, first and middle initials) retrieves all available records. Nevertheless, by searching as described we obtained a more certain identification of the physician.

To estimate the quality of research by a particular physician, we employed two measures. Our main indicator for research quality is a physician’s average citation per publication and our secondary indicator for research quality is the H-index suggested by Hirsch [41]. Average citations per publication is a relatively clean measure of research impact, though its main weakness is its vulnerability to single peaks. The H-index complements our primary research quality measure as it is considered a consistent and systematic indicator of research impact and quality according to developing scientometric literature [42–43]. Yet, by construction the H-index is highly correlated with the quantity of publications, and it suffers from non-linearity in the sense that, at high levels of H-index, a marginal increase is becoming very difficult to achieve (the number of citations required to obtain the H+1 index is constantly increasing at the margin). We hence choose the average citations per publication to be our main quality indicator and the H-index as the secondary one, for robustness purposes. We obtained both average citations and H-index indicators of each physician from the Web of Science, an online academic citation index that covers over 12,000 journals worldwide. Here too, we searched by typing the physician’s surname, first name and middle initial into the “author field,” enabling us to retrieve an average number of citations per item and a personal H-index for publications since 2006. This search procedure might seem limiting. However since 2006, most journals in the three chosen specializations specify authors’ surname and first name, and hence the probability for missed papers is negligible and nonsystematic. Publications not categorized as “medical research” were excluded from the retrieval process. Physicians who had common names, raising the possibility that publications by more than one physician would be retrieved (e.g., publications listed under both ophthalmology and orthopedics), were removed from the sample. At last, both the quality measures we employed reflect the quality of recent research activity carried out by physicians while also maintaining enough variation to conduct statistical analysis (CV = 0.61 and 0.56 for the average citations and H-index, respectively). Overall, the names of 30 physicians were removed from the sample (about 0.7%), yielding an effective sample of 4,330 physicians.

### Quality of medical care

We based our key dependent variable—Index of Hospital Quality (IHQ)—on the 2011–2012 *U*.*S*. *News & World Report*’s “Best Hospitals” ranking, applied at the department level for each of the three specializations. This ranking system is viewed by many physicians and public policy makers as one of the most sophisticated, accurate, and influential means of assessing hospital quality [44–46]. It is based on a comprehensive quality assessment theoretical paradigm, reflecting performance in three intertwined dimensions of healthcare: structure, process, and outcomes [47]. The IHQ is expressed as a composite score between 0 and 100 (with the top-ranked hospitals receiving the highest score), and it incorporates dimensions such as survival rates, success in keeping patients safe, and reputation among physicians. As for the other dimensions of the ranking, they are based on data from multiple sources such as the annual survey database of the American Hospital Association (for a more comprehensive review of the development and use of the IHQ see the “Best Hospitals” ranking’s website [48]). As a separate proxy for quality of care, we also used a sub-dimension of the IHQ index—namely, survival rate. Survival rate refers to hospital’s success at keeping patients alive. It is judged by comparing the number of Medicare inpatients with certain conditions who died within 30 days of admission in the recent three years with the number expected to die given the severity of illness. This measure of hospital quality is composed of four tiers, where the highest survival rate is represented by tier 1 and the lowest by tier 4.

Finally, we collected several relevant covariates known to influence the quality of medical care in hospitals. These are often used in the literature [36], [44] and are described and summarized in Table 1.

**Table 1:** Descriptive statistics of variables employed in the regression analyses.

Variable	Description	Mean	SD	Min	Max
Score^(1)^	Department’s IHQ, 2011–2012	32.85	15.96	8.80	100.00
Survival^(2)^	Department’s patient survival rate–a sub-dimension of IHQ, 2011‒2012	1.88	0.82	1.00	400
Average ^(3)^ citations ^a^	Average number of citations per physician per article, averaged for each department	10.16	6.23	1.07	35
Average citations ^b^	Median of average citations per physician, averaged for each department	6.78	4.66	0.9	31
Average citations ^c^	Average number of citations per physician per article, averaged for each department after eliminating from the sample newly recruited physicians	10.53	7.40	1.26	58.16
H-index ^a^ ^(3)^	H-index since 2006, averaged for each department	4.48	3.08	0.75	14.53
H-index ^b^	Median H-index for each department since 2006	3.18	2.40	0.5	13.5
H-index ^c^	H-index since 2006, averaged for each department after eliminating from the sample newly recruited physicians	4.49	3.10	0.5	17.61
Avg. publications ^a^ ^(4)^	Average no. of publications per physician per year, averaged for each department since 2002	1.43	0.87	0.18	4.38
Avg. publications ^c^	Average no. of publications per physician per year, averaged for each department since 2002, after eliminating from the sample newly recruited physicians	1.87	0.85	0.33	5.62
Physicians ^(5)^	Number of physicians at each department in each hospital	29.05	11.38	2.00	68.00
For-Profit^(6)^	= 1 if the hospital is a for-profit organization	0.30			
Staffed beds^(6)^	Number of patients’ beds in the hospital (in hundreds)	6.80	3.49	1.65	22.86
Clinical services^(6)^	Number of clinical services provided by the hospital	35.03	5.17	21.00	44.00
Length of stay^(7)^	Average number of days of hospitalization	6.27	0.80	4.43	7.92
Median age^(7)^	Median age of 3 geographically closest zip codes to the hospital	34.18	2.97	28.83	41.00
Median income^(7)^	Median income of 3 geographically closest zip codes to the hospital in current thousands of US dollars	40.87	13.25	22.95	83.58
Net profit/loss per physician ^(6)^	Hospital’s total net profit/loss per physician for 2010 in current thousands of US dollars	99.82	213.38	-52.58	1408.11

Sources

(1), (2) 2011–2012 *U*.*S*. *News & World Report*’s “Best Hospitals” ranking

(3) Web of Science database, data collected during 2012

(4) Pubmed database, data collected during 2012

(5) Departments’ websites, data collected during 2012

(6) http://www.ahd.com/freesearch.php, 2011 data

(7) http://www.ahd.com/freesearch.php and United States census bureau, 2010 data.

*IHQ, Index of Hospital Quality

(a) Refers to the simple average of the related figure for each department.

(b) Refers to the median average of the related figure for each department.

(c) Refers to the simple average of the related figure, figure for each department, after eliminating from the sample newly recruited physicians.

### Econometric concerns

Some econometric concerns worth attention. First, note that IHQ, the dependent variable is a complex construct based on both objective data, namely; *structure* (which refers to resources that relate directly to patient care), *outcome* (risk-adjusted mortality rates) and the *process* component of care delivery (which has to do with patient safety) on the one hand, and subjective data, namely “reputation” which is based upon a survey among 200 physicians, on the other hand. As IHQ factors in a reputation component, it may raise concern over endogeneity as reputations might be related to research quality, which is our main independent variable. Note however that for the three specializations chosen in this study, IHQ ranking depends heavily on objective data (i.e. *structure*, *outcome*, and *process*). Moreover, in the survey where physicians were asked to list the hospitals they consider to be the best in their specialization, there was no reference to physicians’ research performance in the process that determines the reputation score. Also, *U*.*S*. *News* conducts a statistical adjustment to keep a small number of hospitals with very high reputational scores from swamping the rest of the field in the final rankings.

Of more concern is the possibility of self-selection; as the assignment of physicians to hospitals cannot be assumed of random nature, a suspicion arises that better researchers may be drawn to hospitals that are in better positions to provide research funding. If such matching exists than the positive correlation between quality of healthcare and various measures of research quantity and quality could just be an artifact of the match between skill and wealth. This problem can be best dealt with by including hospital’s revenue per physician (with a 1-year lag to avoid simultaneity) in the regressions. Given the *ceteris paribus* nature of a regression analysis, this allows one to measure the impact of the independent variables (volume and quality) on the dependent variables (IHQ) holding the level of hospital wealth constant.

Thirdly, a problem of endogeneity may arise if the quality of medical care and the volume and quality of research are determined jointly. This concern can be dismissed, however, because physicians’ research volume and quality as employed here are measures that accumulate over several years. Moreover, accounting for the fact that research outputs published on any given year are usually the result of distant past efforts, simultaneity should not be present.

Lastly, since by construction, high values of the H-index cannot be achieved if the number of publications is small, we were not surprised to find strong positive correlation between the H-index and the average number of publications (Spearman’s rank correlation coefficient is 0.72, p<0.001). Yet, despite this correlation, in most regressions collinearity did not hinder significance tests, hence we felt safe to use the H-index in its original values. That said, in order to grant more credence to our results we also obtained a “clean” measure of quality from the original H-index, by means of running an auxiliary regression, where we regressed the H-index on the number of publications. The residuals from this regression are, by construction, uncorrelated with the number of publications, and are therefore a cleaner measure of quality. However, as the results obtained with the residuals are similar to those obtained by using the original H-index, we do not report them here.

## Results and Discussion

**Fig 1:**
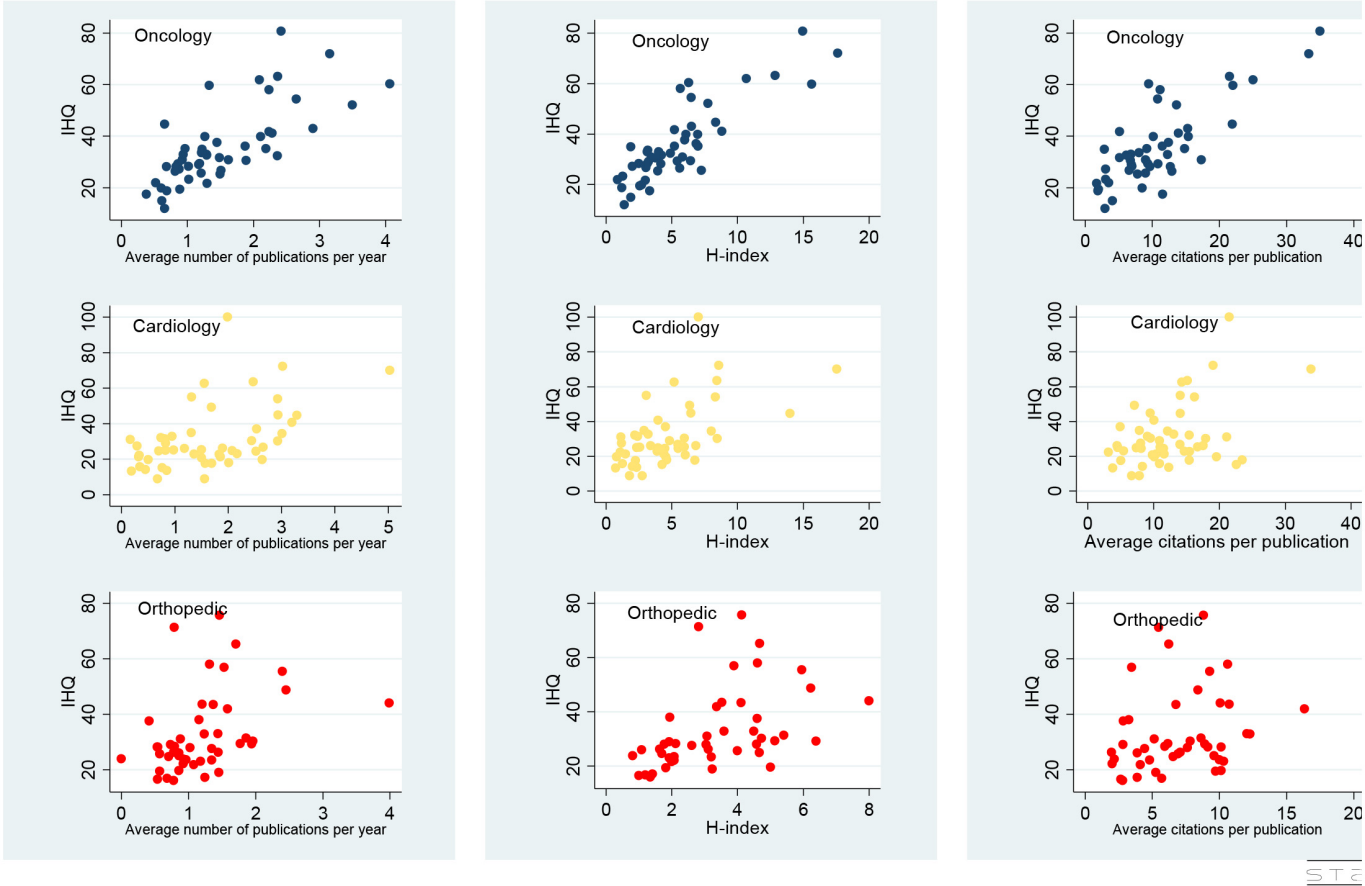
Scatter plots between IHQ and research output measures by specialization. Research output measures include: average citations per publication, H-index, average number of publications per year.

Fig 1 below presents the observed correlations between our key dependent and independent variables, for each specialization. The figure demonstrate that in general there exists a positive correlation between each of the quality/quantity research measures and IHQ. However, the “noise” that is apparent in Fig 1 and the multiple outliers, warrants a more rigorous statistical approach that controls for the possible intervention of other variables. Another notable feature that arises from Fig 1 is the different nature through which IHO is associated with all research quality and quantity measures in the three specializations. This calls for a statistical analysis that employs interaction terms, rather than estimating an average effects.

We now turn to our primary statistical analyses. We employed two models that examined the effects of volume and quality of physicians’ research at the department level on their department’s quality of clinical care—one model for each of our dependent variables, IHQ and survival rate. For the first dependent variable IHQ which is a continuous variable, we capitalized on the panel structure of our dataset and used a fixed-effects model with clustered errors where the effects are the specializations (Hausman specification test suggests rejecting the null hypothesis, i.e., that a random effects model is preferred due to greater efficiency).

The empirical equation estimated is:

$$\begin{array}{l} IHQ_{j}=\alpha_{0}+\alpha_{1}\;for\;Profit{\;}_{j}+\alpha_{2}\;Staffed\;beds_{j}+Length\;of\;stay_{j}+\alpha_{4}\;Median\;income_{j}+\\ \alpha_{5}\;Median\;age_{j}+\alpha_{6}\;Physicians{\;}_{ij}+\alpha_{7}hospital\;income\;per\;physition\;i+\alpha_{8}\;Research\;volume_{j}+\\ \alpha_{9}Research\;volume\;*\;Cardiology{\;}_{j}+\alpha_{10}\;Research\;volume\;*\;Oncology{\;}_{j}+\alpha_{11}\;Research\;quality+\\ \alpha_{12}\;Research\;quality\;*\;Cardiology{\;}_{j}+\alpha_{13}\;Research\;quality\;*\;Oncology+\beta_{i}+\varepsilon_{ij} \end{array}$$

The dependent variable is the IHQ of specialization *i* in hospital *j*. The variables *for-Profit*, *Staffed beds*, *Length of stay*, *Median income*, *Hospital income per physician*, and *Median age*, are control variables at the hospital level. The variable *Physicians*_ij_, represents the number of physicians in specialization *i* in hospital *j*. The interaction terms: Research volume_*_ *Cardiology*_j_, Research quality_*_ *Cardiology*_j,_ Research volume_*_ *Oncology*_j,_ and Research quality*Oncology_j_ aimed at capturing the differential effects of research quality and volume on the specializations Cardiology and Oncology, respectively, relative to the omitted specialization, Orthopedic. Finally, α_k_, k = 1,..,13 is a vector of parameters representing the impact of the independent variables on the dependent variable, βi is a vector of fixed-effects for the three specializations and ɛ_ij_ is the error term.

Table 2 presents the results of two fixed effects regressions. For both regressions the dependent variable is IHQ, whereas Eq.(1) provides the results of the regression that employs the average citation as a proxy for research quality, and Eq.(2) provides the results of the regression that employs our secondary research quality measure, H-index. All other variables remain the same across the two regressions. For ease of presentation and interpretation only the calculated elasticities are provided followed by their standard error.

**Table 2:** Calculated Elasticities based on a Fixed-effects regression with clustered errors, Dependent variable: index of hospital quality (IHQ).

Eq.(1) Avg. citations^(1)^		Eq. (2) H-index^(1)^	
Avg. publications	0.082	Avg. publications	-0.077
	(0.080)		(0.060)
Avg. publications*cardiology	0.053***	Avg. publications*cardiology	0.060**
	(0.016)		(0.016)
Avg. publications*oncology	0.092**	Avg. publications*oncology	0.120**
	(0.037)		(0.033)
Avg. Citations	0.158***	H-index	0.325***
	(0.016)		(0.016)
Avg. Citations*cardiology	0.020**	H-index*cardiology	-0.064***
	(0.007)		(0.013)
Avg. Citations*oncology	0.001	H-index*oncology	-0.032***
	(0.023)		(0.008)
For-profit	-0.059**		-0.059***
	(0.030)		(0.018)
Staffed beds	-0.013		-0.036
	(0.060)		(0.048)
Length of stay	0.650***		0.643**
	(0.191)		(0.194)
Median income	-0.178		-0.149
	(0.130)		(0.113)
Median age	-0.033		-0.128***
	(0.235)		(0.216)
Physicians	0.199*		0.207**
	(0.118)		(0.101)
Net income/loss per physician	0.147**		0.137***
	(0.034)		(0.028)
N = 145	R^2^ = 0.631	N = 145	R^2^ = 0.637

Analyses were conducted in STATA version 13.1, Elasticities are calculated at means.

*(1)* Delta Method standard errors in parentheses

* *p* < 0.10

** *p* < 0.05

*** *p<0*.*001*

As noted, in both regressions the coefficients of the non-interacted terms: *Avg*. *publications*, *Avg*. *citations* and *H-index* refer to the effect of these variables on the IHQ of the omitted specialization (Orthopedics in this case). The coefficients of the interacted terms (Average publications*cardiology, Average publications*Oncology, Average Citations*cardiology, Average Citations*Oncology, H-index*cardiology and H-index*Oncology) refer to the effect of *Avg*. *publication*, *Avg*. *citation* and *H-index* in the specializations Oncology and Cardiology on IHQ relative to their effect in the omitted specialization-Orthopedics. The findings suggest that volume of research has a significant positive effect on clinical excellence, in particular for the specializations Cardiology and Oncology. For example, *ceteris paribus*, an increase of 10% in research volume (*Avg*. *publications*) in Oncology is correlated with an average increase of 0.92% and 1.2% in the IHQ according to Eq.(1) and Eq.(2) respectively, whereas in Cardiology, the same increase in research volume in is correlated with an average increase of 0.53% and 0.6% in the IHQ according to Eq.(1) and Eq.(2) respectively. However, the results show that holding research volume constant, a rise in research quality has a greater effect on clinical performance for all specializations. For example, a rise of 10% in the *Avg*. *citations* in Orthopedics correlates with 1.58% rise in IHQ, 1.78% in Cardiology, and 1.58% in Oncology. For the H-index regression (right column), an increase of 10% in Orthopedics’ H-index correlates with 3.25% increase in the IHQ, 2.65% increase for Cardiology, and 2.93% increase for Oncology.

It is noteworthy that while the distributions of our quality indicators—*Avg*. *citations* and *H-index*—are right skewed for many departments, we still adhere to the use of the average statistic for the following reason: it appears that in majority of cases there is a large portion of new/non-tenured researchers with very low quality figures, and a small portion of senior researchers with high quality figures. We therefore believe, based on conversations with senior physicians/researchers that these relatively high quality researchers are the “tone givers” and the quality benchmarks, and therefore the average better represents the department’s quality of research. Still, for robustness we also conducted the same analysis using medians instead of averages. The results appear in Table 3, and as can be seen, they remain qualitatively similar: while research volume has positive and significant effect in most cases, quality is associated with greater impact on the dependent variable IHQ.

**Table 3:** Calculated Elasticities based on a Fixed-effects regression with clustered errors, dependent variable: index of hospital quality (IHQ).

Eq.(1) Avg. citations^(1)^		Eq.(2) H-index^(1)^	
Avg. publications	0.043	Avg. publications	-0.06*
	(0.057)		(0.025)
Avg. publications*cardiology	0.027**	Avg. publications*cardiology	0.091***
	(0.006)		(0.003)
Avg. publications*Oncology	0.107**	Avg. publications*Oncology	0.109***
	(0.034)		(0.022)
Median Avg. Citations	0.182***	Median H-index	0.205***
	(0.027)		(0.046)
Med. Avg. Citation*cardiology	0.029*	Med. H-index*cardiology	-0.039***
	(0.016)		(0.014)
Med. Avg. Citations*Oncology	-0.035**	Med. H-index*Oncology	-0.035***
	(0.011)		(0.005)
For-Profit	-0.092**		-0.072***
	(0.031)		(0.022)
Staffed beds	0.005		0.026
	(0.110)		(0.086)
Length of stay	0.898		0.751***
	(0.147)		(0.214)
Median income	0.124		0.135
	(0.096)		(0.130)
Median age	-1.143***		-1.034**
	(0.343)		(0.356)
Physicians	0.391*		0.450**
	(0.223)		(0.163)
Net income/loss per Physician	0.208***		0.201***
	(0.064)		(0.063)
N = 145	R^2^ = 0.626		R^2^ = 0.608

Analyses were conducted in STATA version 13.1, Elasticities are calculated at means.

*(1)* Delta Method standard errors in parentheses

* *p* < 0.10

** *p* < 0.05

*** *p<0*.*001*

Note that, as in Table 2, the coefficients of the interacted terms (volume interacted with Cardiology and Oncology and quality interacted with both specialties) are measured relative to the coefficients of the un-interacted terms for volume and quality. These latter terms reflect the impact of research volume and quality, respectively, on IHQ at the omitted specialization (Orthopedics)

We now move on to test the model with the second dependent variable: survival rate, which is an ordered categorical variable ranked from 1 to 4. For this purpose we used an ordered logistic regression.

Because our data included only four observations at the lowest survival rate ranking (i.e., tier 4), they were merged into category 3. For simplicity of exposition and interpretation, we reversed the order of the variable. Also, we included the covariate *clinical services*, which was not included in the previous model because it is used to construct the IHQ variable. Due to space limits this regression was ran only for our main quality measure; average citations, for the secondary measure, due to high collinearity between the H-index and avg. papers, this regression did not yield significant results. Table 4 reports the calculated elasticities, i.e., the change in the probability to be ranked top tier (highest level of survival rate) as each predictor increases by 1% (or from 0 to 1 in the case of a dummy variable).

**Table 4:** Generalized ordered logistics regression with clustered errors. Dependent variable: survival rate (reversed).

Independent Variables^(2)^	Elasticities^(1)^	Delta Method Standard Errors
Avg no. publications	0.022	0.095
Avg no. publications*cardiology	0.076	0.066
Avg no. publications*Oncology	0.189***	0.032
Avg. Citations	0.267***	0.084
Avg. Citations *cardiology	-0.107	0.075
Avg. Citations * Oncology	0.023***	0.021
For-Profit	-0.284*	0.167
Oncology	0.022	0.042
Cardiology	0.177**	0.060
Clinical services	2.123*	1.182
Length of stay	0.313	1.182
Median age	-2.464**	1.010
Median income^(^	-0.712**	0.346
Physicians	-0.096	0.211
Net profit/loss per physician	-1.883	1.295

Analyses were conducted in STATA version 13.1, using the gologit2 module [49]; an advanced version of the generalized ordered logit model [50] for ordinal dependent variables. The gologit model relaxes the proportional odds assumption and allows the effects of the explanatory variables to vary with the point at which the categories of the dependent variable are dichotomized.

N = 145, Log pseudo likelihood = -107.89, Pseudo R^2^ = 0.188

Wald test indicates that the final model does not violate the proportional odds/ parallel lines assumption.

***P<0.01

**P<0.05

*P<0.1

(1) Calculated at means.

(2) The variable *staffed beds* was omitted from this analysis because, given the sample size, the Ordered logistic regression is less resilient than the OLS regression for number of regressors.

(3) The *gologit*2 command, an advanced version of the generalized ordered logit model [50] for ordinal dep. Vars. The gologit model relaxes the proportional odds assumption and allows the effects of the explanatory variables to vary with the point at which the categories of the dependent variable are dichotomized. It also offers several additional powerful options such as a straightforward calculation of elasticities.

The results in Table 4 are in line with those in Table 2; research volume has positive and significant effect on the dependent variable, in particular in Oncology. More specifically, a 10% increase in the number of publications correlates with 1.89% increase in the probability to be ranked in the top tier. Yet, research quality as measured by average citations has a greater effect. For Orthopedics and Cardiology, an increase of 10% in the average citations increases the probability to be ranked top tier by 2.67% on average, and by 2.9% in Oncology.

As a final robustness check, we also run the same regressions after eliminating from the sample physicians who did not publish at all. We assume that these physicians have graduated from medical school not long before the time the survey was conducted and hence they have not yet realized their potential as researchers (S2 Table).

## Discussion

Understanding the relationship between research and practice is a persistent and difficult problem for policy makers and scholars who work in professional schools, such as business, engineering, medicine, education, and public administration. Per the latter, although one of professional schools’ key missions is developing knowledge that can be translated into skills that advance the practice of the professions, this mission often remains an elusive ideal [3].

We try to contribute to the debate over the link between academic research and practice by shedding light on the dilemma whether the volume or the quality of academic research provides a more valid measure of research contribution to practice [10], [15], [17]. This dilemma is relevant for many academic disciplines and for society at large. Although the debate remains unsolved in many cases, the fact that physicians at university hospitals produce both research output and medical care of measurable quality presents a unique opportunity to measure the differential effect of high-quality versus high-volume biomedical academic research on medical practice, thereby offering a partial answer to the debate.

In this study, we empirically demonstrated that high-quality research is *significantly correlated with* quality of medical care, as measured by IHQ and survival rate. While we also show that high-volume research may also correlates with quality of medical care, this effect is significantly weaker than the correlation of high-quality research with quality of medical care. For any given level of research volume an increase of 1% in high-quality research contributes more to quality of medical care than an increase in 1% of high-volume research. Our results provide empirical evidence for the claim that in most cases high-quality research, is associated with more value to practice [14–16], [51]. Our interpretation of the results is as follows: owing to the synergy between biomedical research and medical practice, and based on the assumption that high-quality research uses more meticulous standards, more advanced methods and more cutting-edge techniques, it should produce better results in terms of practical outcome than high-volume, often low-quality research. Moreover, we maintain that the actual positive association between the quality of biomedical research and the quality of medical care is even stronger than the apparent results because in principle it is reasonable to assume that those institutions that conduct high-quality research often attract the most severe medical cases.

Little information is available on the contribution of biomedical academic research to the quality of medical care. Previous research has focused primarily on the relationship between hospital teaching status and health care quality. Most of these studies suggested that clinical care outcomes in teaching hospitals are better than in non-teaching hospitals [52], although these findings are also under debate [53–54]. To the best of our knowledge, the association between biomedical research and hospitals’ quality of care has been examined in only one empirical study [36] which focused on one category (cardiovascular) and adopted a basic analytical approach (Spearman's rank correlation tests). The authors of that study reported a moderate link between physicians’ research productivity (e.g., numbers of publications and citations) and hospital-level risk-adjusted mortality ratio for congestive heart failure and acute myocardial infarction, suggesting that increased research productivity may lead to better healthcare. This result points to one theoretically relevant linkage between biomedical research and practice, perhaps the most intuitive one. However, bibliometric research on the more general relationship between academic research and university/academic unit ranking shows that the reality is more complex; A variety of research publication strategies exist, and different strategies may yield different consequences [55]. Our study indeed demonstrates the different consequences of high-volume versus high-quality research productivity strategies.

### Limitation of our study

Several caveats of our research should be mentioned. The first is related to the fact that we are able to measure only the direct effect between research and practice. Although research volume by itself has *relatively smaller effect* on quality of medical care, it may have an indirect effect. More specifically, in reality research volume and research quality are correlated and it could be argued that a combination of some level of volume with an emphasis on quality research is optimal [19], [35]. Unfortunately, in most cases resource constraints force scientists (and physicians) to choose one publication strategy over the other.

A second caveat of this research, which opens the door for future related studies, has to do with the measurement of quality. Quality of research is a complex concept, the measuring of which entails more sophisticated methods than a simple scalar measures such as the H-index. Although we used different measures of research quality, the scatter plot matrix (S1 Fig) demonstrates a positive correlation between the two measures (especially in Oncology). Future research may therefore consider the use of other research quality measures. For example, the percentage or number of papers published in the top 10% journals of the field may be a meaningful marker for quality. While the debate on how to measure research quality rests outside the scope of this study, we still feel that obtaining similar results while using two different quality measures renders more consistency to our methodological approach.

Finally, due to the cross sectional nature of our data, the existence of causal relationship between biomedical research quality and quality of medical care cannot be inferred. The latter requires a panel data with time series.

## Conclusions

The management of research—from selection of ideas and projects to choice of the average time spent on a research project—is clearly incentive-dependent. Our results imply that incentives should be directed at scientists, physicians, or academic departments that yield research publications of high impact. This view finds some empirical support in the work of Harzing [39], who demonstrates (although not only in the biomedical research area) that the Australian government’s performance evaluations of academic institutions showed a sharp decline in research impact during the period in which evaluation emphasized volume rather than quality of publications. Our study also corresponds with Azoulay, Zivin, and Manso [56] who showed that incentive schemes that reward long-term success, tolerate early failure and give its appointees greater freedom to experiment and take more risky and innovative scientific projects, are more conductive to scientific breakthroughs.

Our research may have far-reaching policy implications for the creation of incentive schemes aimed at scientists, the public funding of academic research, and policy making at the government and regulatory levels. More specifically, it conveys implications for physicians engaged in biomedical academic research, for the public funding of biomedical research, and for health-policy making at government and hospital levels. An important policy implication of this research concerns the need to initiate an adequate publicly funded system for evaluation of quality and impact standards, according to which the performance of academic departments and individual scientists will be assessed and financially supported. Some countries, notably the UK and Australia, already employ such systems.

In the specific context of biomedical research, many hospital physicians are under great pressure to publish (or perish) and have to juggle between patients, research, teaching, and administrative work. This reality often drives them to compromise on the quality of biomedical research in favor of volume of publications. The vast majority of biomedical research papers are indeed published in low-impact journals [31]. Moreover, the pressure to publish sometimes even leads to scientific fraud [32, 37]. Our findings may help persuade health policymakers, hospital managers, and physicians to consider a shift in the current approach by designing and providing the necessary incentives for physicians to use their scarce time in the most valuable way, that is to say by carrying out high-quality research, even at the expense of lower volume.

## Supporting Information

S1 FigCorrelation between H-index and Avg. Citations (standardized) by specialization.Standardized scores for Average citations and Average H-index were calculated at the department level in hospital j for each specialization i as follows: $\frac{\text{Average}\;(\text{score})\;\text{j}–\text{mean}(\text{score})\text{i}}{\text{Standard dev}.(\text{score})\text{i}}$
(TIF)

S1 Table List of university hospitals studied. (DOCX)

S2 Table Calculated Elasticities based on a Fixed-effects regression with clustered errors, dependent variable: index of hospital quality (IHQ). Quality and quantity indicators calculated after omitting newly recruited physicians’ data.
(DOCX)
