# Supplementary figures and images for: From Research to Practice: Which Research Strategy Contributes More to Clinical Excellence? Comparing High-Volume versus High-Quality Biomedical Research
Source: PLoS One. 2015 Jun 24;10(6):e0129259. doi: 10.1371/journal.pone.0129259 (PMC4480880; doi:10.1371/journal.pone.0129259)

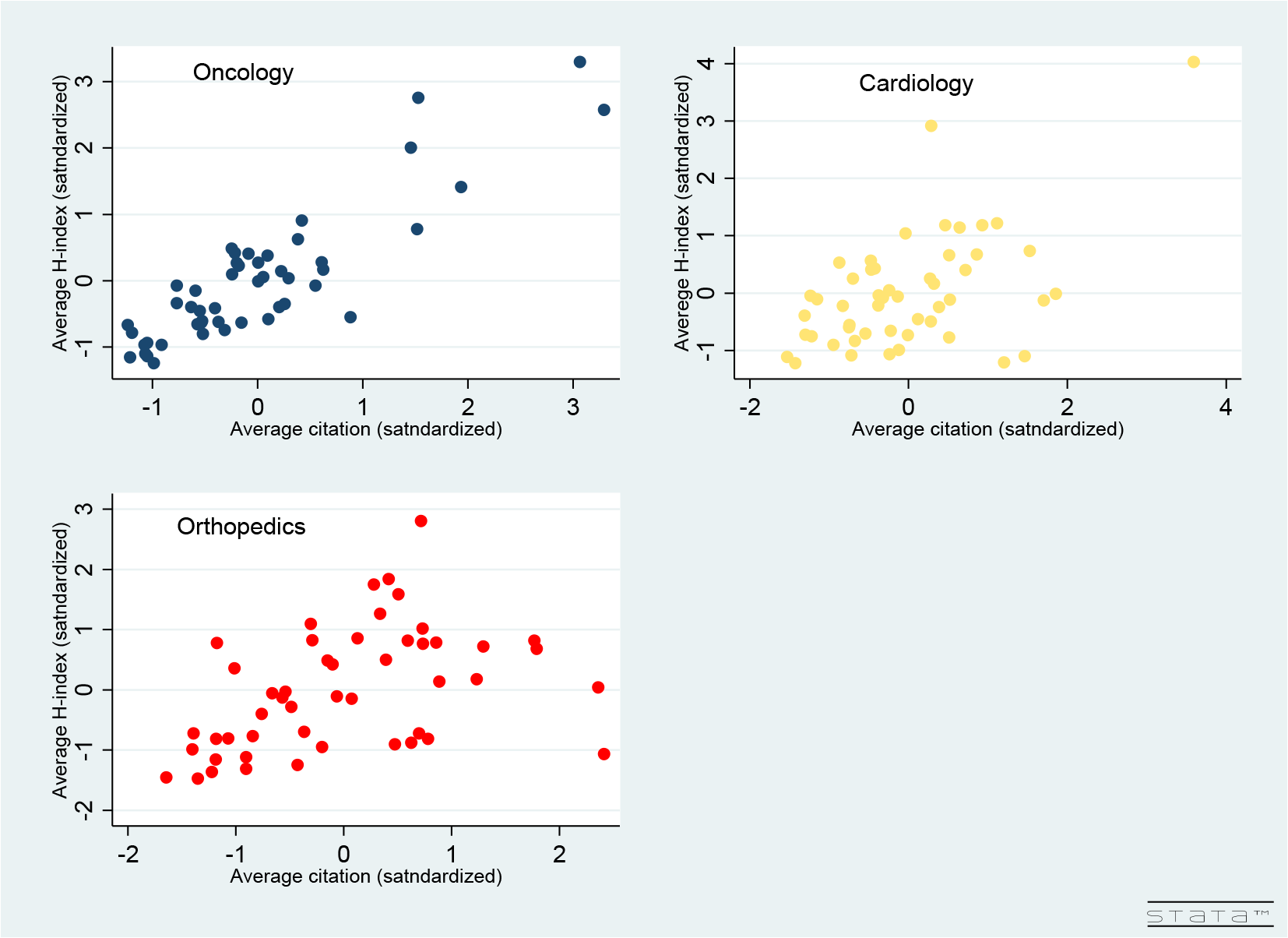

Supplement: S1 Fig — Standardized scores for Average citations and Average H-index were calculated at the department level in hospital j for each specialization i as follows: Average(score)j–mean(score)iStandard dev.(score)i (TIF) [file pone.0129259.s001.tif]
