# Supplementary material for: From Research to Practice: Which Research Strategy Contributes More to Clinical Excellence? Comparing High-Volume versus High-Quality Biomedical Research
Source: PLoS One. 2015 Jun 24;10(6):e0129259. doi: 10.1371/journal.pone.0129259 (PMC4480880; doi:10.1371/journal.pone.0129259)
Supplement: S1 Table — (DOCX) [file pone.0129259.s002.docx]

| **Hospital** | | **City** | **State** |
| --- | --- | --- | --- |
| Cleveland Clinic | Cleveland | | OH |
| Johns Hopkins Hospital 125 years | Baltimore | | MD |
| Massachusetts General Hospital | Boston | | MA |
| New York-Presbyterian University Hospital of Columbia & Cornell network of 16 | NYC | | NY |
| Brigham and Women's Hospital | Boston | | MA |
| Ronald Reagan UCLA Medical Center Los Angeles | Los Angeles | | CA |
| Duke University Medical Center | Durham | | NC |
| Hospital of the University of Pennsylvania Philadelphia | Philadelphia | | PA |
| University of Michigan Hospitals and Health Centers | Ann Arbor | | MI |
| Northwestern Memorial Hospital | Chicago | | IL |
| UPMC-University of Pittsburgh Medical Center | Pittsburgh | | PA |
| Vanderbilt University Medical Center | Nashville | | TN |
| Loyola University Medical Center | Maywood | | IL |
| University of Alabama Hospital at Birmingham | Birmingham | | AL |
| Hahnemann University Hospital | Philadelphia | | PA |
| University of Kansas Hospital | Kansas City | | KS |
| Harper University Hospital Detroit | Detroit | | MI |
| Hackensack University Medical Center | Hackensack | | NJ |
| Wake Forest University Baptist Medical Center | Winston-Salem | | NC |
| University of California San Francisco Medical Center | San Francisco | | CA |
| Stony Brook University Hospital | Stony Brook | | NY |
| Robert Wood Johnson University Hospital | New Brunswick | | NJ |
| University of Minnesota Medical Center | Minneapolis | | MN |
| Baylor University Medical Center | Dallas | | TX |
| University of Wisconsin Hospital and Clinics | Madison | | WI |
| University Hospitals Case Medical Center | Cleveland | | OH |
| Oregon Health and Science University | Portland | | OR |
| Dartmouth-Hitchcock Medical Center | Lebanon | | NH |
| University of California Davis Medical Center | Sacramento | | CA |
| University of Virginia Medical Center | Charlottesville | | VA |
| University of Rochester Medical Center | Rochester | | NY |
| University Hospital of Brooklyn-SUNY Downstate Medical Center chain of 6 hospitals | Brooklyn | | NY |
| Loma Linda University Medical Center | Loma Linda | | CA |
| St. Louis University Hospital | Saint Louis | | MO |
| North Shore University Hospital Manhasset network of 6 | Manhasset | | NY |
| Memorial University Medical Center | Savannah | | GA |
| University of Kentucky Chandler Hospital | Lexington | | KY |
| Penn State Milton S. Hershey Medical Center | Hershey | | PA |
| Boston Medical Center | Boston | | MA |
| Staten Island University Hospital network of 6 | Staten Island | | NY |
| George Washington University Hospital | Washington | | DC |
| University of Utah Health Care | Salt Lake City | | UT |
| West Virginia University Hospitals | Morgantown | | WV |
| University Community Hospital | Tampa | | FL |
| North shore University Hospital (network of 6) | Evanston | | IL |
| OSF St. Francis Medical Center (network of 4) | Peoria | | IL |
| Gunderson Lutheran La Crosse | La Crosse | | WI |
| SUNY Upstate Medical University | Syracuse | | NY |
| University Medical Center | Lubbock | | TX |
| University of Missouri Health Care Columbia | Columbia | | MO |
