# Supplementary material for: From Research to Practice: Which Research Strategy Contributes More to Clinical Excellence? Comparing High-Volume versus High-Quality Biomedical Research
Source: PLoS One. 2015 Jun 24;10(6):e0129259. doi: 10.1371/journal.pone.0129259 (PMC4480880; doi:10.1371/journal.pone.0129259)
Supplement: S2 Table — Quality and quantity indicators calculated after omitting newly recruited physicians’ data. (DOCX) [file pone.0129259.s003.docx]

| Calculated Elasticities based on a Fixed-effects regression with clustered errors^(1,2)^, dependent variable: index of hospital quality (IHQ) | | | | | | | | |  |  |  |
| --- | --- | --- | --- | --- | --- | --- | --- | --- | --- | --- | --- |
|  | | Eq.(1) Avg. citations | | | Eq. (2) H-index | | | | | |  |
|  | | |  | | |  | | | |  | |
| Avg. publications^(3)^ | 0.301**  (0.128) | | | Avg. publications^(3)^ | | | 0.085  (0.094) | | | | |
| Avg. publications*cardiology^(3)^ | 0.012  (0.047) | | | Avg. publications*cardiology^(3)^ | | | 0.017**  (0.026) | | | | |
| Avg. publications*Oncology^(3)^ | 0.063  (0.063) | | | Avg. publications*Oncology^(3)^ | | | 0.097 **  (0.045) | | | | |
| Avg. Citations^(3)^ | 0.310**  (0.132) | | | H-index^(3)^ | | | 0.267***  (0.014) | | | | |
| Avg. Citations *cardiology^(3)^ | -0.146  (0. 701) | | | H-index *cardiology^(3)^ | | | -0.011**  (0.005) | | | | |
| Avg. Citations *Oncology^(3)^ | -0.067  (0.048) | | | H-index *Oncology^(3)^ | | | -0.042**  (0.016) | | | | |
| For-profit | -0.048  (0.035) | | |  | | | -0.054***  (0.018) | | | | |
| Staffed beds | -0.012  (0.087) | | |  | | | 0.025  (0.048) | | | | |
| Length of stay | 0.664***  (0.313) | | |  | | | 0.478**  (0.070) | | | | |
| Median income | -0.195  (0.132) | | |  | | | -0.173  (0.150) | | | | |
| Median age | 0.178  (0.358) | | |  | | | -0.070  (0.110) | | | | |
| Physicians | 0.212**  (0.092) | | |  | | | 0.213**  (0.096) | | | | |
| Net income/loss per physician | 0.148***  (0.035) | | |  | | | | 0.127***  (0.029) | | | |

N=145 R^2^= 0.588 R^2^=0.629

1. Delta Method standard errors in parentheses
2. Elasticities are calculated at means
3. Quality and quantity indicators calculated after omitting newly recruited physicians’ data

* *p* < 0.10, ** *p* < 0.05, *** *p<0.001*
